# *Lycium barbarum* polysaccharides as prebiotics prevent colorectal cancer liver metastasis in non-alcoholic fatty liver disease by modulating gut microbiota–*FGF21-PI3K-AKT* axis

**DOI:** 10.3389/fphar.2026.1735434

**Published:** 2026-03-13

**Authors:** Shuai Zhao, Zhenyao Tan, Jiaxin Suo, Yang Bu

**Affiliations:** 1 Department of Hepatobiliary Surgery, General Hospital of Ningxia Medical University, Yinchuan, China; 2 The First Clinical Medical College, Ningxia Medical University, Yinchuan, China; 3 People’s Hospital of Ningxia Hui Autonomous Region, Ningxia Medical University, Yinchuan, China

**Keywords:** acylcarnitine metabolism, colorectal cancer liver metastasis, gut microbiota, Lycium barbarum polysaccharides, non-alcoholic fatty liver disease, short-chain fatty acids

## Abstract

**Introduction:**

Colorectal cancer liver metastasis (CRLM) is the leading cause of death in colorectal cancer, and nonalcoholic fatty liver disease (NAFLD) promotes CRLM. *Lycium barbarum* polysaccharides (LBPs), bioactive metabolites of the traditional medicinal plant *Lycium barbarum* L, inhibit the progression of colorectal cancer and NAFLD by regulating gut microbiota composition. However, their roles in preventing CRLM under NAFLD conditions remain unclear. This study aimed to investigate the preventive effect of LBPs on liver metastasis of colorectal cancer in the context of NAFLD and explore its potential mechanisms.

**Methods:**

An NAFLD mouse model was established, followed by prophylactic oral administration of LBPs by gavage for 28 days before splenic injection of MC38 colorectal cancer cells to establish liver metastasis. Pseudo-germ-free mice combined with fecal microbiota transplantation were constructed to explore the role of the gut microbiota in the preventive effect of LBPs on CRLM. Gut microbiota and fecal short-chain fatty acids were analyzed by 16S rRNA sequencing and liquid chromatography–mass spectrometry. Spearman’s correlation analysis was used to explore the correlation between bacterial genera and liver lipid metabolism indicators. Serum non-targeted metabolomic profiling and transcriptomic analysis of CRLM cells were performed to elucidate metabolic and molecular mechanisms.

**Results:**

Under NAFLD conditions, LBPs markedly reduced hepatic metastatic burden, liver weight, and liver-to-body weight ratio. LBPs ameliorated hepatic lipid metabolism and restored colonic barrier integrity in NAFLD mice. The gut microbiota was identified as a critical mediator of LBPs-induced protection against CRLM, and depletion of the microbiota completely abrogated the anti-metastatic effects of LBPs. LBPs enhanced microbial diversity and richness, enriched of short-chain fatty acid-producing bacterial genera, such as *Cryptobacteroides, Evtepia, and Bacteroides*-H, and elevated colonic butyrate levels. Metabolomic profiling revealed reduced serum acylcarnitines and increased organic acids. Transcriptomic profiling showed upregulation of fibroblast growth factor 21, activation of the PI3K-AKT signaling pathway, and promotion of epithelial–mesenchymal transition in colorectal cancer cells, while LBPs reverse these changes.

**Discussion:**

LBPs prevent CRLM associated with NAFLD by modulating the gut microbiota, enhancing butyrate production, improving hepatic metabolic homeostasis, and suppressing prometastatic signaling pathways. These findings highlight LBPs as promising preventive agents against CRLM in the setting of metabolic liver disease.

## 1 Introduction

Colorectal cancer (CRC) ranks as the third most commonly diagnosed malignancy and the second leading cause of cancer-related mortality worldwide. Distant metastasis is a major contributor to the high mortality observed in patients with CRC, with the liver representing the most frequent site of metastatic stress (Wang et al., 2024). Approximately 50% of patients with CRC develop liver metastases during the course of the disease, and 80%–90% of these cases are initially deemed unresectable. Even among patients who undergo curative-intent liver resection, postoperative recurrence rates remain high, reaching up to 60% (Chinese College of Surgeons, 2025). Therefore, the development of novel strategies to prevent and treat colorectal cancer liver metastases (CRLM) is critical for improving long-term survival and reducing mortality in patients with CRC.

Non-alcoholic fatty liver disease (NAFLD) is the most prevalent chronic liver disorder worldwide, with a steadily increasing prevalence, particularly in China. NAFLD is recognized not only as an independent risk factor for synchronous CRLM (Lv and Zhang, 2020) but also as a predictor of intrahepatic recurrence following resection of liver metastases (Hamady et al., 2013). Consistent with the “seed and soil” hypothesis, the NAFLD-associated hepatic microenvironment provides a permissive niche for the engraftment and growth of metastatic tumor cells. Specifically, hepatocyte-derived extracellular vesicles in NAFLD facilitate CRLM by creating an immunosuppressive hepatic microenvironment (Wang Z. et al., 2023). Additionally, the lipid-rich microenvironment characteristic of NAFLD upregulates fatty acid synthase in metastatic CRC cells, thereby activating endogenous palmitate biosynthesis, promoting tumor cell stemness, and facilitating CRLM(Zhang et al., 2024). Moreover, NAFLD is characterized by a chronic inflammatory microenvironment in which elevated proinflammatory mediators—including tumor necrosis factor alpha, interleukin-6, and activation of the nucleotide-binding oligomerization domain-like receptor family C member 4 inflammasome—facilitate adhesion, proliferation, and angiogenesis of circulating CRC cells within hepatic sinusoidal endothelial cells (Ohashi et al., 2019). Collectively, these findings suggest that modulating lipid metabolic disorders, attenuating hepatic inflammation, and improving immune dysfunction may mitigate the pro-metastatic role of NAFLD in CRC.

*Lycium barbarum* L has been used for thousands of years in traditional Chinese medicine to prevent and treat metabolic and liver-related diseases, including diabetes mellitus, hyperlipidemia, and hepatitis (Yu et al., 2023). In the Compendium of Materia Medica, *Lycium barbarum* L is described as having therapeutic functions of “nourishing liver and kidney” and ”brightening eyes” (Xiao et al., 2022). *Lycium barbarum* polysaccharides (LBPs), the principal bioactive metabolite of *Lycium barbarum* L, function as prebiotics and have been shown to improve hepatic lipid metabolism and alleviate hepatic inflammatory responses (Feng et al., 2022; Xiao et al., 2022). LBPs are classified as non-starch polysaccharides that directly interact with the gut microbiota, thereby influencing host gut metabolic homeostasis (Wang et al., 2025). On one hand, in a mouse model of hepatocellular carcinoma, LBPs markedly suppressed tumor growth by increasing T-cell populations in peripheral blood, lymph nodes, and tumor tissues, reducing regulatory T cells, promoting CD8^+^ T-cell infiltration, and alleviating T-cell exhaustion (Deng et al., 2018). On the other hand, LBPs remodel gut microbiota composition, promote short-chain fatty acids (SCFAs) production, enhance fatty acid *β*-oxidation in hepatocytes, and improve the lipid-enriched hepatic microenvironment. (Feng et al., 2022; Li et al., 2022). Importantly, SCFAs inhibit the occurrence and progression of CRC by promoting immune cell differentiation, downregulating proinflammatory mediators, and suppressing tumor-induced angiogenesis (Gomes et al., 2023). Therefore, for NAFLD, LBPs may exert a preventive effect against CRLM through prebiotic-mediated mechanisms.

In the present study, we demonstrate for the first time the preventive efficacy of LBPs against liver metastasis in CRC. This effect was evaluated in a mouse model of CRLM established by intra-splenic injection of tumor cells in the context of NAFLD. Mechanistically, our findings reveal that LBPs prevent CRLM by regulating the gut microbiota, promoting butyrate production, and improving the hepatic microenvironment. This study offers a novel therapeutic perspective for preventing CRLM in the context of NAFLD.

## 2 Materials and methods

### 2.1 Materials and reagents

The LBPs (product no. B20460) were purchased from Shanghai Yuanye Bio-Technology Co., Ltd. (Shanghai, China). The product was tested in compliance with the Pharmacopoeia of the People’s Republic of China (Quality Inspection Report No. KS375929).The polysaccharide content in LBPs is 50.19%. The Fibroblast growth factor 21(FGF21)(A23463) primary antibody was purchased from ABclonal Biotechnology (Wuhan, China). The PI3 Kinase (13666S), Phospho-PI3 Kinase (p-PI3K) (4228S), AKT (4691S), and phospho-AKT (p-AKT) (4060S) primary antibodies were purchased from Cell Signaling Technology (Boston, United States). Vimentin (BF8006), E-cadherin (BF0219), and N-cadherin (AF5239) primary antibodies were purchased from Affinity Biosciences LTD (Jiangsu, China). Zonula occludens-1 (ZO-1)(ab221547), Occlidin (ab216327), and Claudin (ab211737) primary antibodies were purchased from Abcam (Cambridge, United Kingdom). *β*-Actin (BS6007MH) primary antibody was purchased from Bioworld (Nanjing, China), and HRP-conjugated Goat Anti-Rabbit IgG(H+L) (SA00001-2) and HRP-conjugated Goat Anti-Mouse IgG(H+L) (SA00001-1) secondary antibodies were purchased from Proteintech (Wuhan, China). Vancomycin hydrochloride (V105495), neomycin sulfate (N109017), metronidazole (M109874), and ampicillin sodium (A105483) were purchased from Aladdin (Shanghai, China). Other chemical products used were at least analytical grade.

### 2.2 Extraction, detection, and monosaccharide composition of LBPs

This study utilized Ningxia *Lycium barbarum* L, which are included in the Pharmacopoeia of the People’s Republic of China. *Lycium barbarum* L are the dried, mature fruits of Lycium barbarum, a plant belonging to the genus Lycium within the family Solanaceae. Extraction and characterization of LBPs were performed in accordance with the ConPhyMP standard (Michael et al., 2022). LBPs were extracted using hot-water extraction followed by ethanol precipitation. Dried Ningxia *Lycium barbarum* L were cleaned to remove impurities, thoroughly washed, drained, and ground into powder. Water was added at liquid-to-solid ratios of 10:1–15:1 (v/w), and the mixture was refluxed at 90 °C–95 °C for 2 h. The extraction procedure was repeated three times. The combined filtrates were concentrated under reduced pressure to a relative density of 1.10–1.15 at 60 °C. Ethanol was then added to achieve a final concentration of 60%–70% (v/v) to precipitate polysaccharides. Proteins were removed using the Sevag method. The solution was reconcentrated and vacuum-dried at 55 °C–60 °C. The dried extract was ground and passed through a 100-mesh sieve to obtain LBPs. The polysaccharide content of *Lycium barbarum* L was determined using ultraviolet-visible spectrophotometry.

Monosaccharide composition analysis was conducted following the Chinese National Standard (GB/T 44739–2024) using high-performance liquid chromatography. Briefly, an appropriate amount of sample was weighed and dissolved in 3.5 mL of distilled water, followed by ultrasonication for 30 min. One milliliter of the sample solution was accurately transferred into an ampoule, and 4 mL of trifluoroacetic acid was added. The ampoule was sealed and acidified in an oven at 110 °C for 4 h. After cooling, the ampoule was opened, and the Hydrolysate was dried at 105 °C. Methanol (3 mL) was added, and the sample was dried again. The residue dissolved in 2 mL ammonia solution, filtered through a 0.22 μm membrane, and reserved for derivatization.For derivatization, 0.1 mL of sample solution was mixed with 0.1 mL of 0.5 mol/L 1-phenyl-3-methyl-5-pyrazolone-methanol solution in methanol and incubated at 70 °C for 30 min. The reaction mixture was evaporated to dryness under nitrogen, followed by the addition of 1 mL of methanol, and evaporation. The residue was dissolved in 1.0 mL of water, and excess derivatization reagent was removed by extraction with chloroform until the chloroform phase became colorless. The aqueous state was filtered through a 0.22 μm membrane and analyzed using an Agilent 1100 HPLC system equipped with a diode-array detector (Agilent Technologies, Santa Clara, United States).

### 2.3 Animal experiment design

Animal experiments were conducted in accordance with the Guidelines for the Care and Use of Laboratory Animals published by the National Institutes of Health (NIH Publication No. 8023, 1978) and were approved by the Ningxia Medical University Institutional Animal Care and Use Committee (IACUC-NYLAC-2024-073). Male C57BL/6J mice aged 6–8 weeks (20–23 g) were purchased from the Laboratory Animal Center of Ningxia Medical University (License No.: SCXK Ning. 2015-0001) and were housed in a specific pathogen-free environment with a 12-h light/12-h dark cycle. All mice had free access to food and water. The mice model of NAFLD was established as described in reference (Wang Z. et al., 2023). Specifically, mice were randomly divided into two groups: the control group (Control), which was fed a normal diet (Beijing Kexiaoxili Feed Co., Ltd., Beijing, China, n = 11); and the high-fat diet group (NAFLD) (60 kcal% from fat, PD6001, SYSE Bio, Jiangsu, China, n = 12). After 12 weeks, three mice from each group were sacrificed, and liver tissues were examined by histopathological sections according to the “Guidelines for the Diagnosis and Treatment of Non-alcoholic Fatty Liver Disease” to determine whether the NAFLD model was successfully established.

### 2.4 Cell culture

MC38 (mice CRC cell line, CX0158) was obtained from Doctor De Biotechnology Co., Ltd. (Wuhan, China) and grows in RPMI-1640 medium containing 1% penicillin-streptomycin and 10% fetal bovine serum. The cells are placed in a incubator at 37 °C with 5% CO_2_. Before injection, the cells are resuspended in phosphate buffer saline.

### 2.5 Drug intervention and intrasplenic injection

After the NAFLD model was successfully established, the NAFLD group mice were randomly divided into two groups: NL group (NAFLDLBP group) (fed with high-fat diet and oral gavaged with LBPs at a dose of 200 mg/kg (Li et al., 2022; 2023; Liang et al., 2023) once a day, n = 5); N group (NAFLD group) (fed with high-fat diet and oral gavaged with the same volume of normal saline once a day, n = 4). The Control group mice were randomly divided into two groups: L group (fed with normal diet and oral gavaged with LBPs at a dose of 200 mg/kg,once a day, n = 4); C group (Control group) (fed with normal diet and oral gavaged with the same volume of normal saline once a day, n = 4). The feeding continued for 4 weeks.

After the intragastric administration of each group of mice was completed, MC38 cells (5 × 10^5^ cells per mice) were injected into the spleen under isoflurane inhalation anesthesia and the mice were continued to be fed for 2 weeks. Fecal samples were collected within 24 h before euthanasia. After all the experiments were completed, the mice were fasted for 12 h and then euthanized to collect blood and other tissues. The body weight and liver weight of each group of mice, as well as the tumor burden in the liver, were recorded. The left liver lobes from each group of mice were dissected for hematoxylin and eosin (H&E) staining. The proximal colon segments were used for immunohistochemical analysis. All samples, except for the immediately centrifuged blood, were stored at −80 °C.

### 2.6 Detection of biochemical indicators in mice serum

Following anesthesia induction in mice, blood was collected via cardiac puncture and allowed to stand for 30 min. Serum was subsequently separated by centrifugation at 3000 rpm for 15 min. Quantitative analysis of serum lipid parameters (including Total cholesterol (TC), Triglyceride (TG),High-Density lipoprotein cholesterol (HDL), and Low-Density lipoprotein cholesterol (LDL)) and hepatic function indicators (Aspartate Aminotransferase (AST) and Alanine Aminotransferase (ALT)) was performed using a fully automated biochemical analyzer (LST008α, Hitachi High-Technologies Corporation, Tokyo, Japan).

### 2.7 Immunohistochemistry

Fresh left liver lobe and colon specimens were collected, rinsed with physiological saline, and preserved in 4% paraformaldehyde. The tissues were subsequently embedded in paraffin and sectioned for H&E staining and immunohistochemical (IHC) analysis. For histological examination, sections were deparaffinized and subjected to H&E staining. For IHC analysis, tissue sections were deparaffinized, rehydrated, and underwent antigen retrieval in citrate buffer.Briefly, after antigen retrieval, sections were blocked with 10% normal goat serum at room temperature for 30 min. They were then incubated overnight at 4 °C with primary antibodies (anti-ZO-1, anti-Occludin, anti-Claudin) diluted 1:100 in 10% normal goat serum. After phosphate buffer saline wash, sections were incubated with a secondary antibody for 1 h at room temperature, followed by a 30-min incubation with ABC reagent. Following an additional phosphate buffer saline wash, diaminobenzidine chromogen was applied and developed for 2–7 min at room temperature based on signal intensity. Finally, slides were counterstained with hematoxylin, dehydrated, and mounted. Histological images were acquired using an Aperio LV1 slide scanner (Leica, Germany).

### 2.8 Construct pseudo-germ-free mice using a mixture of antibiotics (ABX)

Upon successful establishment of the NAFLD model, pseudo-germ-free mice were prepared according to Reference (Li et al., 2024) prior to LBPs intervention or fecal microbiota transplantation (FMT). Specifically, an antibiotic mixture solution containing metronidazole (1 g/L), ampicillin sodium (1 g/L), vancomycin hydrochloride (0.5 g/L), and neomycin sulfate (1 g/L) was administered. mice in the FMT group and the NLA group received Oral gavage ABX solution (200 μL once daily) for 7 days before FMT or drug intervention. ABX administration was discontinued in the FMT group upon initiation of FMT, whereas the NLA group continued to receive ABX throughout the LBPs intervention period.

### 2.9 Fecal microbiota transplantation (FMT)

The FMT procedure was performed in accordance with the protocol described in reference (Bokoliya et al., 2021). Briefly, on the day of transplantation, fresh fecal samples (80 mg/kg/day) were collected from N-group donors (n = 5) or NL-group donors (n = 5), resuspended in 0.9% sterile saline (100 mg/mL), homogenized, and centrifuged at 800 rpm for 3 min. The supernatant was collected for FMT. Recipient mice with substantially depleted commensal microbiota were randomly allocated into two groups (n = 5 per group): one group received transplantation of microbiota from N-group donors (FN group), and the other received microbiota from NL-group donors (FNL group). Receptor mice were administered 200 μL via oral gavage.

### 2.10 16S ribosomal RNA gene sequencing

Genomic DNA was extracted from fecal samples using the MagBeads FastDNA Kit for Soil (Cat: 116564384C1, MP Biomedicals). The quality of the isolated DNA was assessed using a Nanodrop NC2000 spectrophotometer (Thermo Scientific, Waltham, MA, United States) and agarose gel electrophoresis (LIUYI Biotechnology, Beijing, China), followed by storage at −20 °C until further use. Bacterial community structure was characterized by 16S ribosomal RNA gene sequencing. The V3-V4 hypervariable region of the bacterial 16S rRNA gene was amplified via polymerase chain reaction (PCR) using the forward primer (5′-ACT​CCT​ACG​GGA​GGC​AGCA-3′) and reverse primer (5′-GGACTACHVGGGTWTCTAAT-3′). Amplified fragments were sequenced on the Illumina platform (Novaseq-PE250) using paired-end sequencing at Personal Biotechnology Co., Ltd. (Shanghai, China). PCR amplification was performed in a 25 μL reaction mixture containing Q5® High-Fidelity DNA Polymerase (NEB, Ipswich, MA, cat.# M0491L), 10 μmol/L of each primer, and 2 μL of DNA template. The thermal cycling protocol consisted of initial denaturation at 98 °C for 30 s, followed by 25 cycles of denaturation at 98 °C for 30 s, annealing at 50 °C for 30 s, and extension at 72 °C for 30 s. Primers were incorporated into the template to synthesize DNA, resulting in the accumulation of amplified fragments over each cycle. A final extension step was carried out at 72 °C for 5 min, and the products were subsequently held at 4 °C.

### 2.11 Non-targeted metabolite sequencing of serum samples

Serum samples stored at −80 °C were thawed and vortexed. A 50 μL aliquot was transferred to a 2 mL centrifuge tube, mixed with 200 μL of pre-cooled methanol:acetonitrile (1:1, v/v), and vortexed for 30 s. The mixture was then frozen at −20 °C for 30 min, followed by centrifugation at 12,000 rpm and 4 °C for 10 min. A 200 μL portion of the supernatant was collected and vacuum-dried to completeness. The residue was reconstituted in 150 μL of 50% methanol (containing 5 ppm 2-chlorophenylalanine) with vortexing for 30 s, then centrifuged again under the same conditions. The resulting supernatant was passed through a 0.22 μm filter membrane, and the filtrate was transferred to a detection vial.

All samples were analyzed using a Thermo Vanquish Flex ultra-high-performance liquid chromatography system coupled with a Thermo Orbitrap Exploris 120 high-resolution mass spectrometer. Separation was performed on an ACQUITY UPLC HSS T3 column (100Å, 1.8 µm, 2.1 mm × 100 mm) maintained at 40 °C, with a flow rate of 0.4 mL/min and an autosampler temperature of 8 °C. The injection volume was 2 μL. The mobile phases consisted of 0.1% formic acid in water (phase A) and acetonitrile containing 0.1% formic acid (phase B) for both positive and negative ionization modes. The gradient elution program was as follows: 0–1 min, 5% B; 1–4.7 min, linear increase to 95% B; 4.7–6 min, 95% B; 6.1–8.5 min, 5% B.

Data acquisition in data-dependent acquisition mode for both positive and negative ions was controlled by Xcalibur software (version 4.7, Thermo) using a heated electrospray ionization source. The spray voltage was set to 3.5 kV for positive and −3.0 kV for negative mode, with sheath gas at 40 arb, auxiliary gas at 10 arb, capillary temperature at 320 °C, and auxiliary gas heater temperature at 300 °C. Full-scan mass spectrometry were acquired at a resolution of 60,000 over a mass range of 70–1000 m/z, with an automatic gain control target of “Standard” and maximum injection time of 100 ms. The top 4 most intense ions were selected for fragmentation with a dynamic exclusion time of 4 s. Tandem mass spectrometry spectra were recorded at a resolution of 15,000 using higher-energy collisional dissociation at 30% normalized collision energy, with automatic maximum injection time and standard Automatic gain control target.Raw data files were processed using Mass Spectrometry Data Independent Analysis software (version 4.9.221218) for peak extraction, alignment, filtering, and metabolite identification. Peaks not detected in more than 50% of quality control samples were filtered out, and missing values were imputed using the software’s gap-filling algorithm followed by normalization. Metabolite identification was conducted by matching against the PerSonalbio Next-Generation Metabolomics Database provided by Shanghai Personal Biotechnology Co., Ltd.

### 2.12 Detection of SCFAs

Measure appropriate amounts of pure standard samples of acetic acid, propionic acid, butyric acid, isobutyric acid, valeric acid, isovaleric acid, caproic acid, and 2-ethylbutyric acid (internal standard) to prepare a standard curve. Place 20 mg of feces sample into a 2 mL centrifuge tube. Add two steel balls and 800 μL of extraction reagent (containing internal standard). Vortex for 60 s. Place into a tissue grinder and grind at 55 Hz for 60 s. Repeat once. Centrifuge at 4000 *g*, centrifuge at 4000 *g* for 10 min at 10 °C; Take 40 μL supernatant, add 20 μL 200 mM 3-nitrophenylhydrazine, mix well; Add 20 μL 120 mM 1-Ethyl-3-(3-dimethylaminopropyl)carbodiimide hydrochloride-6% pyridine solution, mix well; Incubate in a thermostat at 40 °C, shaking at 1200 rpm for 30 min; After reaction, cool on ice for 3 min; centrifuge at 12,000 g, 4 °C for 10 min; take 50 μL supernatant, add 150 μL formic acid water (v/v, containing 0.1% formic acid) to a final volume of 200 μL, vortex for 60 s; Centrifuge at 12,000 g, 4 °C for 10 min; transfer supernatant through a 0.22 μm filter membrane and bottle the filtrate. Determine single-chain fatty acid mass using a Triple Quad™ 6500+ system and ExionLC-AE (AB Sciex Pte. Ltd., Marlborough, U.S.A.), based on the standard curve of ES peak area.

### 2.13 Transcriptome sequencing analysis of liver metastases

Total RNA was isolated from liver metastasis samples using Trizol Reagent (Invitrogen, United States), followed by genomic DNA removal with DNase I (TaKaRa, Shiga, Japan). After assessing RNA concentration, purity, and integrity, total RNA samples with a minimum quantity of ≥1 μg were selected for library preparation. Polyadenylated mRNA was enriched using Oligo (dT) magnetic beads and subsequently fragmented randomly via ion-mediated cleavage in the presence of divalent cations, employing the NEBNext Ultra II RNA Library Prep Kit for Illumina (New England Biolabs Inc; Ipswich, Massachusetts, United States). First-strand cDNA synthesis was performed using fragmented mRNA as template and random oligonucleotides as primers. The double-stranded cDNA was purified, followed by end repair, adenylation of 3′ends, and adapter ligation. cDNA fragments ranging from 400 to 500 bp were size-selected using AMPure XP beads, amplified by PCR, and further purified with AMPure XP beads to construct the final library. Library quality was assessed using the Agilent 2100 Bioanalyzer with the Agilent High Sensitivity DNA Kit (Agilent Technologies Inc, California, United States, 5067-4626). The total library concentration was quantified via PicoGreen fluorescence measurement (Quantifluor-ST fluorometer, Promega, Madison, Wisconsin, United States, E6090; Quant-iT PicoGreen dsDNA Assay Kit, Invitrogen, California, United States, P7589), and the effective library concentration was determined by quantitative PCR (StepOnePlus Real-Time PCR Systems, Thermo Scientific, Waltham, Massachusetts, United States). Multiplexed DNA libraries were normalized and pooled in equal volumes. The pooled library was subjected to stepwise dilution and quantification prior to sequencing on an Illumina platform under PE150 mode.

The data filtration criteria primarily include: 1) Removal of sequences with adapters at the 3′-end using fastp (v0.22.0); 2) Exclusion of reads with an average quality score below Q20. Clean reads were aligned to the reference genome in stranded mode using Hierarchical Indexing for Spliced Alignment of Transcripts 2. Mapped reads from each sample were subsequently assembled using StringTie. Differential gene expression analysis between comparison groups was performed with DESeq2 (v1.38.3), with differentially expressed genes identified under the following thresholds: |log2FoldChange| > 1 and adjusted P-value <0.05. Functional enrichment analysis of differentially expressed genes—including Gene Ontology (GO), Kyoto Encyclopedia of Genes and Genomes (KEGG) pathway enrichment, and Gene Set Enrichment Analysis (GSEA)—was conducted using clusterProfiler (v4.6.0).

### 2.14 Western blot

Three mice specimens were randomly selected from each group. Liver metastatic lesions samples (100 mg) were homogenized in lysis buffer to extract total protein, and protein concentration was quantified using a bicinchoninic acid protein assay kit. After separation by sodium dodecyl sulfate–polyacrylamide gel electrophoresis (SDS-PAGE), the protein samples were transferred onto polyvinylidene fluoride membranes. The membranes were blocked with 5% (w/v) skim milk for 2 h, followed by overnight incubation at 4 °C with primary antibodies against FGF21 (1:1000), PI3K (1:1000), p-PI3K (1:1000), AKT (1:1000), p-AKT (1:2000), vimentin (1:1000), E-cadherin (1:1000), and N-cadherin (1:1000). After three 10-min washes with TBST, secondary antibodies were applied and incubated for 2 h. Protein bands were visualized by chemiluminescence detection, and signals were captured using a bioanalytical imaging system. All Western blot analyses were performed in triplicate.

### 2.15 Statistic analysis

Statistical analyses were performed using GraphPad Prism version 10.4.1. All statistical tests were two-sided. Unpaired t-tests were applied for comparisons between two groups, while one-way analysis of variance (ANOVA) was employed for comparisons among multiple groups. Two-way ANOVA was utilized to assess the effects of two or more independent variables between groups. The Spearman correlation analysis was conducted to evaluate the relationship between microbial abundance and hepatic lipid parameters as well as liver function indicators. A p-value of less than 0.05 was considered statistically significant.

## 3 Results

### 3.1 LBPs monosaccharide composition analysis

**FIGURE 1:**
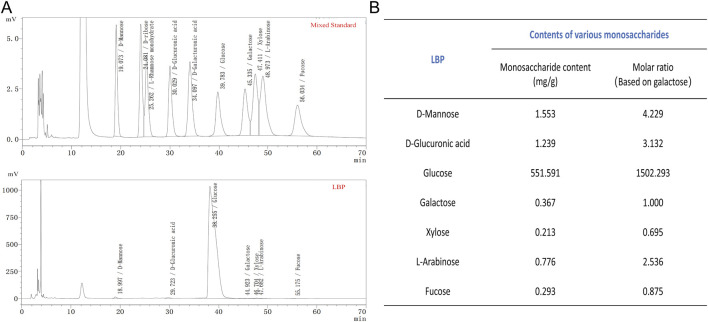
LBPs monosaccharide Composition Analysis. **(A)** HPLC of LBPs. **(B)** The monosaccharide composition of LBPs.

High-performance liquid chromatography (HPLC) analysis of monosaccharide composition yielded the following elution sequence for monosaccharide standards: mannose, ribose, rhamnose, glucuronic acid, galacturonic acid, glucose, galactose, xylose, arabinose, and fucose. Comparative analysis of retention times, peak areas, and calibration curves between LBPs and standards confirmed that LBPs comprised seven monosaccharides: mannose, glucuronic acid, glucose, galactose, xylose, arabinose, and fucose (Figure 1A). The relative content of each monosaccharide is shown in Figure 1B.

### 3.2 LBPs prevent CRLM in the context of NAFLD

**FIGURE 2:**
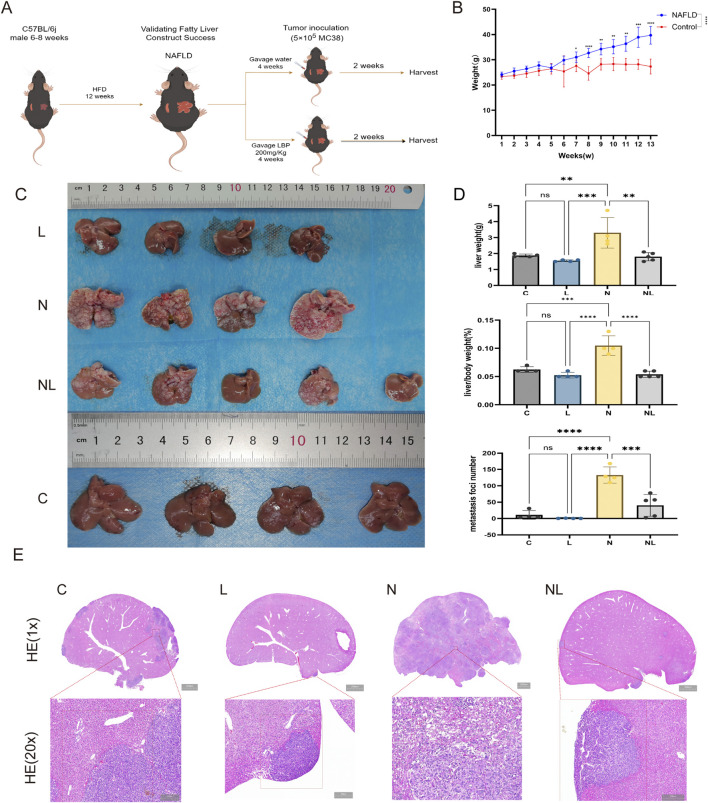
LBPs prevent CRLM in the context of NAFLD. **(A)** Schematic diagram of mice modeling, **(B)** Body weight values of mice at different times. **(C)** Mice liver tissue image. **(D)** Comparison of liver weight, liver-to-body weight ratio, and number of metastatic foci among different groups of mice, **(E)** Representative H&E staining images of the left lobe of the liver from different groups (n = 4, 5). C represents the control group, L represents the LBPs group, N represents the NAFLD group, and NL represents the NAFLD LBP group. *P < 0.05, **P < 0.01, ***P < 0.001, ****P < 0.0001.

A schematic representation of the animal experimental design is shown in Figure 2A. Following 12 weeks of dietary intervention, three mice from each of the Control and NAFLD groups were sacrificed to confirm successful NAFLD model establishment. The NAFLD group gained significantly more weight than the Control group from week 7 onward (Figure 2B). The livers of mice in the NAFLD group were markedly enlarged, pinkish in color, and exhibited blunted edges with a greasy surface (Supplementary Figure A). Liver weight and the liver-to-body weight ratio were significantly increased (Supplementary Figure E). H&E staining showed multiple small balloon-like changes in the liver parenchyma, while Oil Red O staining demonstrated extensive lipid droplets accumulation within hepatocytes (Supplementary Figures B,C). Mice in the NAFLD group also exhibited significant weight gain and metabolic abnormalities, including increased serum TC, TG, and LDL levels (Supplementary Figure D) as well as significant glucose metabolism abnormalities (Supplementary Figure F). These findings confirmed the successful establishment of the NAFLD mouse model.

On the NAFLD background, a CRLM mouse model was established by injecting MC38 cells into the spleen. The number of solid liver metastatic foci in LBPs-treated mice (NL group) was significantly reduced compared with that in the N group. This reduction was accompanied by marked decreases in liver weight and the liver-to-body weight ratio (Figures 2C,D). H&E staining of the left hepatic lobe revealed that the number of metastatic foci in the NL group was lower than that in the N group (Figure 2E).

### 3.3 LBPs ameliorated hepatic lipid metabolism and restored colonic barrier integrity

**FIGURE 3:**
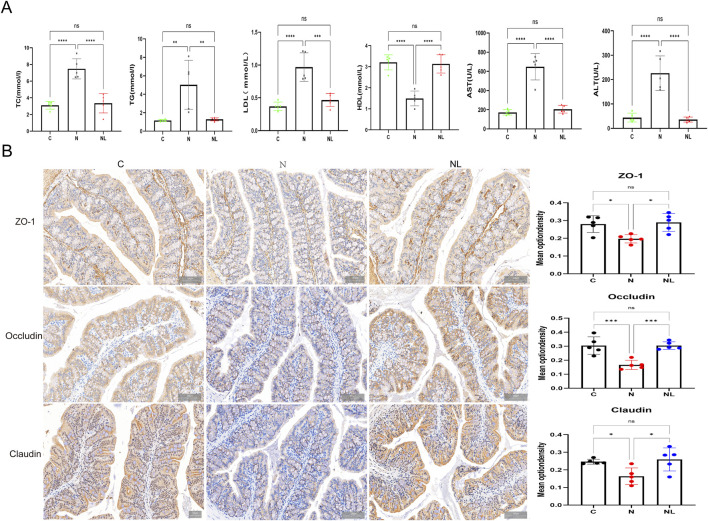
LBPs ameliorated hepatic lipid metabolism and restored colonic barrier integrity. **(A)** The levels of total cholesterol (TC), triglycerides (TG), low-density lipoprotein (LDL), high-density lipoprotein (HDL), aspartate aminotransferase (AST), and alanine aminotransferase (ALT) in the serum of C, N, NL group of mice (n = 5). **(B)** Immunohistochemical method was used to detect the expression levels of ZO-1, Occludin and Claudin proteins in the colons of mice in each group (n = 5). *P < 0.05, **P < 0.01, ***P < 0.001, ****P < 0.0001.

The lipid-rich microenvironment characteristic of NAFLD promotes CRLM (Zhang et al., 2024). Disruption of colonic barrier integrity further exacerbates the lipid-rich microenvironment in NALFD. (Mijra et al., 2023). Therefore, we detected the lipid metabolism indicators in the serum of mice. Serum levels of TG, TC, LDL,AST, and ALT were significantly lower in the NL group than in the N group, while HDL levels were significantly elevated. All parameters recovered to levels comparable to those in the C group (Figure 3A).

Immunohistochemical staining of colonic tissues demonstrated that the expression levels of tight junction proteins ZO-1, Occludin, and Claudin were higher in the NL group than in the N group. These findings suggest that LBPs effectively restored impaired colonic barrier function (Figure 3B).

### 3.4 Gut microbiota serves as a critical mediator of the anti-metastatic effects of LBPs

**FIGURE 4:**
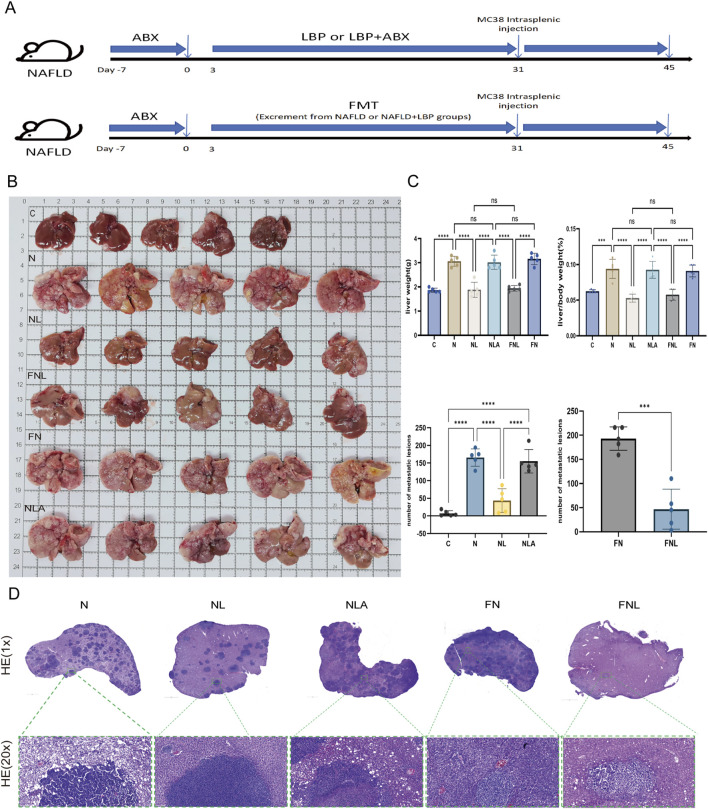
The gut microbiota is the key medium through which LBPs exerts its anti-metastasis effect. **(A)** Schematic diagram of the construction of pseudo-germ-free mice and fecal microbiota transplantation experiments, **(B)** Mice liver tissue image in each group, **(C)** Statistics of liver weight, liver-to-body weight ratio, and the number of metastatic foci in each group. **(D)** Representative H&E staining images of the left lobe of the liver from different groups (n = 5).C represents the control group, N represents the NAFLD group, NL represents the NAFLDLBP group, FNL represents the fecal microbiota transplantation NAFLDLBP group, FN represents the fecal microbiota transplantation NAFLD group, and NLA represents the pseudo-germ-free mice group. *P < 0.05, **P < 0.01, ***P < 0.001, ****P < 0.0001. n = 5/group.

The extremely low bioavailability of LBPs and their reliance on carbohydrate-active enzymes derived from the gut microbiota for decomposition and utilization have been previously established (Ding et al., 2019; Liang et al., 2023),We therefore hypothesized that the protective effects of LBPs against CRLM in NAFLD are mediated through modulation of the gut microbiota. A schematic diagram of the pseudo-germ-free mouse model and FMT procedure is shown in Figure 4A.

Following the depletion of gut microbiota, the liver metastatic burden in the NLA group was significantly increased compared to that in the NL group. Liver weight and liver-to-body weight ratio in the NLA group were comparable to those observed in the N group (Figures 4B,C). H&E staining of the left lobe of the liver revealed that the number of metastatic foci in the NLA group was significantly higher than that in the NL group (Figures 4C,D).

Conversely, mice receiving microbiota transplantation from the NL group (FNL group) exhibited significantly reduced hepatic metastatic lesions compared with mice transplanted with microbiota from the N group (FN group). This reduction was accompanied by lower liver weight and liver-to-body weight ratios (Figures 4B,C). Histological analysis of the left lobe of the liver confirmed a significantly lower number of metastatic foci in the FNL than in the FN group (Figures 4C,D). These findings suggest that the gut microbiota plays a key role in mediating the anti-metastatic effects of LBPs.

### 3.5 LBPs enhance gut microbiota abundance and diversity

**FIGURE 5:**
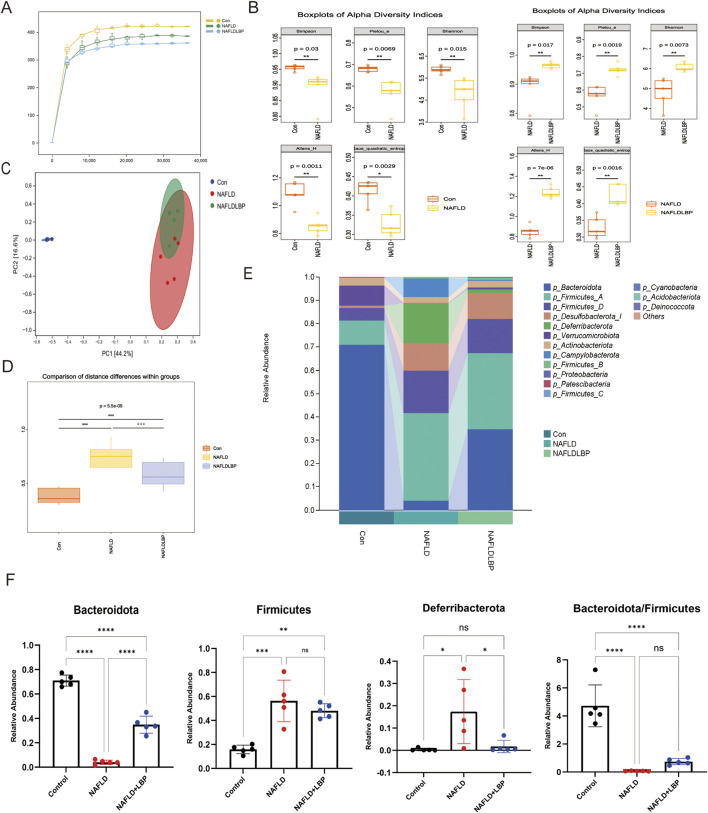
LBPs enhance the abundance and diversity of the gut microbiota. **(A)** Sparse curves of Control, NAFLD, NAFLDLBP group; **(B)** Alpha diversity analysis among different groups; **(C)** Principal Coordinates Analysis (PCoA) illustrating difference in gut microbiota composition among Control, NAFLD and NAFLDLBP group; **(D)** Comparison of microbiome composition differences among the Control Group, NAFLD Group, and NAFLDLBP Group; **(E,F)** The differences in the phylum level of the gut microbiota among each group. *P < 0.05, **P < 0.01, ***P < 0.001, ****P < 0.0001.n = 5/group.

To characterize LBP-induced changes in the gut microbiota, we analyzed the fecal samples from the Control group (C group), NAFLD group (N group), and NAFLDLBP group (NLgroup) using 16S rRNA sequencing technology. Rarefaction curves indicated sufficient sequencing depth across all three groups (Figure 5A). Alpha diversity comparison revealed reduced bacterial richness in the NAFLD group compared with the Control group, whereas bacterial richness was significantly higher in the NAFLDLBP group (Figure 5B). Principal component analysis of bacterial *β*-diversity based on operational taxonomic units indicated significant clustering among the NAFLD, NAFLDLBP, and Control groups, indicating significant differences in microbial community composition. There were also differences between the NAFLD and NAFLDLBP groups (Figure 5C). Intergroup difference testing further confirmed significant microbiota compositional differences among the Control, NAFLD, and NAFLDLBP groups (Figure 5D). At the phylum level, the relative abundance of *Bacteroidota* was lower in the NAFLD group than in the Control group, whereas *Deferribacterota* abundance was higher. LBPs intervention increased *Bacteroidota* abundance and reduced *Deferribacterota* levels. The *Bacteroidota/Firmicutes* ratio was lower in the NAFLD group than in the Control group. In the NAFLDLBP group, the ratio increased, but there was no statistically difference compared with that in the NAFLD group (Figures 5E,F).

### 3.6 LBPs regulate the abundance of bacterial genera and increase butyrate levels in the gut

**FIGURE 6:**
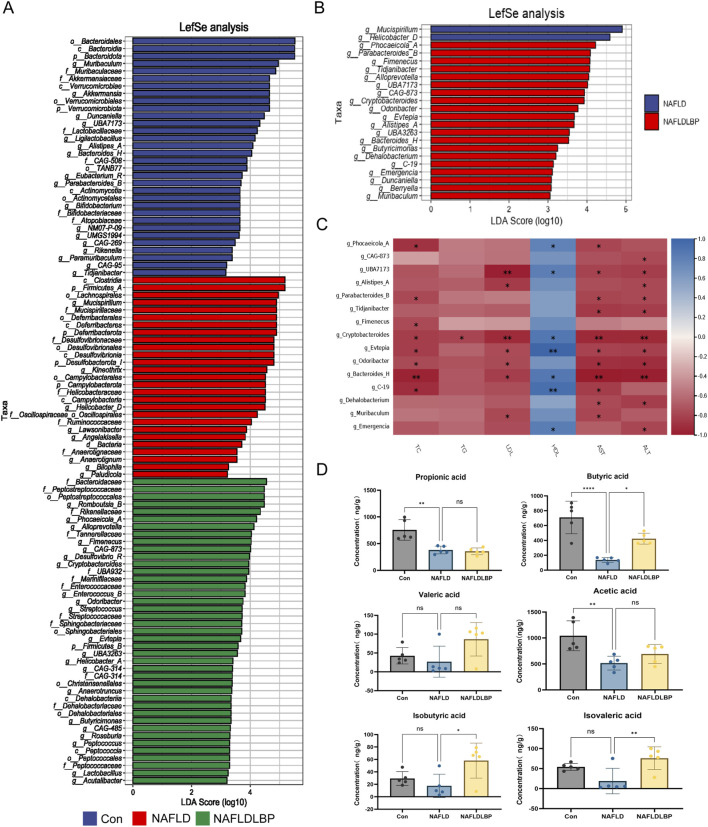
LBPs increase the abundance of specific bacterial genera and the content of butyric acid. **(A)** LEfSe analysis identifies microbial taxa significantly enriched in the Control (blue), NAFLD (red), and NAFLDLBP (green) groups; **(B)** LEfSe analysis of the differences in gut microbiota genus levels between the NAFLD group and the NAFLDLBP group (LDA score threshold of >2 and a significance cutoff of p < 0.05); **(C)** Spearman correlation analysis of the differences in bacterial genera between the NAFLD group and the NAFLDLBP group and the liver lipid metabolism indicators; **(D)** Targeted metabolomics sequencing was used to detect the levels of acetic acid, propionic acid, butyric acid, valeric acid, isobutyric acid and isovaleric acid in the feces of each group of mice.*P < 0.05, **P < 0.01, ***P < 0.001, ****P < 0.0001. n = 5.

Linear discriminant analysis effect size (LEfSe) analysis was performed to identify bacterial taxa with significantly different abundances among groups, using an Linear Discriminant Analysis score threshold of >2 and a significance cutoff of p < 0.05 (Figure 6A). Direct comparison between the NAFLD and NAFLDLBP groups further highlighted distinct microbial alterations induced by LBP treatment (Figure 6B). Specifically, LBP administration significantly increased the relative abundance of *Phocaeicola A, Parabacteroides B, Fimenecus, Tidjanibacter, Alloprevotella, UBA7173, CAG-873, Cryptobacteroides, Odoribacter, Evtepia, Alistipes A, UBA3263, Bacteroides H, Butyricimonas, Dehalobacterium, C-19, Emergencia, Duncaniella, Berryella, and Muribaculum* in the NAFLDLBP group compared to the NAFLD group.

Spearman correlation analysis revealed that *Cryptobacteroides, Evtepia, and Bacteroides H* were negatively correlated with serum TC, LDL, AST, and ALT levels, and positively correlated with HDL levels (Figure 6C). This suggests their potential involvement in modulating hepatic lipid metabolism and liver function.

LBPs are fermented by gut microbiota into bioactive metabolites, particularly SCFAs, which serve as key energy substrates for colonic epithelial cells and regulate liver metabolism via the gut–liver axis (Feng et al., 2022). Targeted metabolomic profiling confirmed that butyrate levels were markedly reduced in the NAFLD group; however, these were significantly restored following LBP intervention (Figure 6D).

### 3.7 LBPs reduce serum acylcarnitine levels and increase organic acid concentrations

**FIGURE 7:**
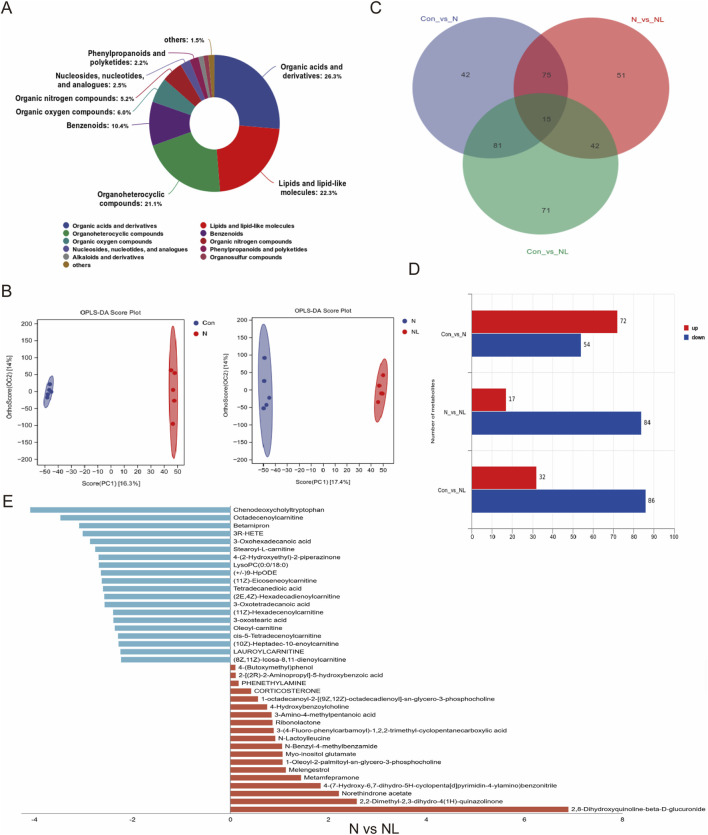
LBPs Reduce Serum Acylcarnitine Levels and Increase Organic Acid Concentrations. **(A)** The proportion of the top 10 categories of metabolites in mice serum; **(B)** Differences in the composition of metabolites among the three groups; **(C)** Venn diagram illustrating the overlap of differentially abundant metabolites across Con vs. N, N vs. NL, and Con vs. NL comparisons. **(D)** Distribution of up- and down-regulated metabolites in pairwise comparisons between Con, N, and NL groups; **(E)** The positive and negative coordinate graph shows the specific information of differential metabolites between the N and NL groups.

To further clarify the specific serum metabolic changes following LBPs intervention in the NAFLD-related CRLM mouse model, we used non-targeted metabolomic analysis to detect the serum of mice in the Con(C groups), N, and NL groups. Identified metabolites were primarily enriched in Organic acids and derivatives (26.3%), lipids and lipid-like molecules (22.3%), and Organoheterocyclic compounds (21.1%) (Figure 7A). Orthogonal partial least squares discriminant analysis demonstrated clear separation among the three groups, indicating significant differences in serum metabolite profiles (Figure 7B). A total of 377 differentially metabolites were detected, with 15 metabolites common to all three groups (Figure 7C). Compared with the Con group, 72 metabolites were increased, and 54 were decreased in the N group. Compared with the N group, 84 metabolites were decreased, and 17 were increased in the NL group (Figure 7D). Differential metabolite analysis revealed that acylcarnitines, including octadecenoylcarnitine, stearoyl-L-carnitine, and oleoyl-carnitine, were reduced following LBP intervention. By contrast, organic acids such as 2-[(2R)-2-aminopropyl]-5-hydroxybenzoic acid and 3-amino-4-methylpentanoic acid were increased (Figure 7E).

### 3.8 Under NAFLD conditions, prophylactic administration of LBPs reverses activation of the FGF21-PI3K-AKT pathway and epithelial-mesenchymal transition in metastatic colorectal cancer cells

**FIGURE 8:**
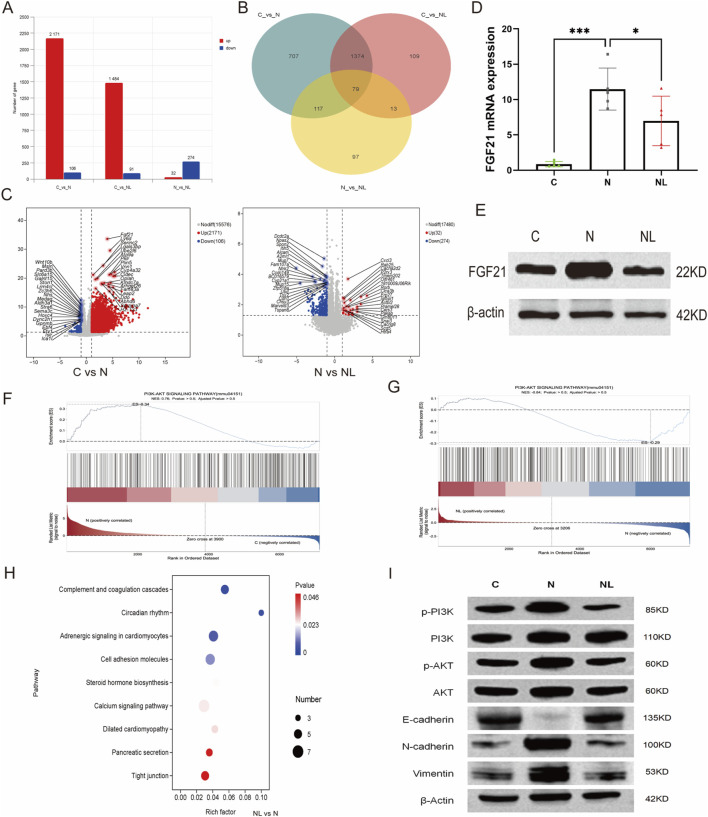
*FGF21-PI3K-AKT* and EMT are suppressed in colon cancer cells metastasized to the liver following LBPs treatment. **(A)** The number of differentially expressed genes among the C, N and NL groups; **(B)** Venn diagram illustrating the overlap of differentially expressed genes across three pairwise comparisons; **(C)** The volcano plot shows the specific information of differentially expressed genes among the three groups; **(D)** Comparison of FGF21 mRNA expression levels among three groups of samples (n = 5); **(E)** Western blot was used to detect the protein level of FGF21 in liver metastases of the three groups; **(F,G)** KEGG-GSEA enrichment analysis plots of the PI3K-AKT signaling pathway among the three groups; **(H)** Bubble chart of the top 20 KEGG pathways of differentially expressed genes between the N group and the NL group; **(I)** Western blot was used to detect the expression levels of proteins in the PI3K-AKT signaling pathway and EMT proteins.*P < 0.05, **P < 0.01, ***P < 0.001, ****P < 0.0001. n = 5.

To explore the effects of LBPs intervention on colorectal cancer cells that metastasized to the liver, we conducted transcriptome sequencing of liver metastases lesions from the C, N, and NL groups. Compared with the C group, 2,171 genes were upregulated, and 106 genes were downregulated in the N group. In the NL group, 1,484 genes were upregulated, and 91 were downregulated compared with C group. Relative to the N group, the NL group had 32 upregulated genes and 274 downregulated genes (Figure 8A). Venn diagram shows 79 genes commonly differentially expressed among C, N, and NL groups (Figure 8B).

Volcano plot analysis demonstrated significant upregulation of *FGF21* in the N group compared with C group (Figure 8C). Both transcript and protein levels of *FGF21* were elevated in the N group, whereas their expression was downregulated following LBP intervention (NL group) (Figures 8D,E). *FGF21* levels are elevated in the blood of colorectal cancer patients and are associated with poor prognosis (Rejali et al., 2024). The PI3K-AKT pathway is one of the most frequently activated signaling pathways in various cancers and is critical in the function of *FGF21* (Guo et al., 2018; Pan et al., 2019; Yu et al., 2021). KEGG-GSEA set enrichment analysis revealed that the PI3K-AKT pathway showed a potential upregulation trend in the NAFLD group. The LBP intervention might lead to the downregulation of the PI3K-AKT pathway (Figures 8F,G). Consistently, protein expression of p-PI3K and p-AKT was elevated in the N group and downregulated following LBP treatment (Figure 8I). KEGG enrichment analysis further revealed that differentially expressed genes between the N and NL groups were enriched in metastasis-related pathways (Figure 8H). Finally, analysis of proteins associated with epithelial-mesenchymal transition in liver metastases at the protein level revealed reduced E-cadherin expression and increased N-cadherin and vimentin expression in the N group, whereas the NL group exhibited the opposite trend. This indicates that LBP intervention reversed the epithelial–mesenchymal transition of CRC cells (Figure 8I).

## 4 Discussion

In traditional Chinese medicine theory, *Lycium barbarum* L.is believed to nourish the liver and kidneys and improve eyesight. Its principal bioactive constituent, LBPs, are currently a major focus of pharmacological research (Yu et al., 2023). LBPs function as a “prebiotic” that regulates gut microbiota, repairs damaged intestinal barrier function, improves liver lipid metabolism, and ameliorates NAFLD (Feng et al., 2022). NAFLD markedly promotes CRLM (Chakraborty and Wang, 2020). Against this background, this study is the first to explore whether LBPs can prevent CRLM under NAFLD conditions. Our results showed that prophylactic oral administration of LBPs significantly reduced the number of metastatic lesions in NAFLD mice and prevented CRLM. Additionally, experiments using pseudo-germ-free mice and fecal microbiota transplantation confirmed that the gut microbiota is the main mediator through which LBPs exert their inhibitory effects on CRLM. LBPs increased gut microbiota richness and diversity, promoted the abundance of *Cryptobacteroides, Evtepia, Bacteroides H*, and related taxa, elevated colonic butyrate levels, improved lipid metabolic disorders associated with NAFLD, and restored colonic barrier integrity. Concurrently, LBPs reduced circulating acylcarnitine levels and reversed epithelial–mesenchymal transition and activation of the *FGF21-PI3K-AKT* signaling pathway in colorectal cancer cells metastasizing to the liver, thereby suppressing CRLM.

A defining feature of NAFLD is lipid metabolic dysregulation, which induces lipotoxicity, remodels the liver microenvironment, and promotes tumor metastasis. The lipid-rich microenvironment characteristic of NAFLD enhances *de novo* palmitate synthesis in metastatic colorectal cancer cells and promotes liver metastasis by stabilizing epidermal growth factor receptor through palmitoylation-dependent membrane localization (Zhang et al., 2024). In the NAFLD mouse model, insulin-like growth factor-1 (IGF-1) expression is increased, leading to activation of signal transducer and activator of transcription 3 and promotion of liver metastasis in colorectal cancer (Strathearn et al., 2020). The knockdown of IGF-1 reduces macrophage infiltration and lowers metastasis in colorectal cancer (Wu et al., 2010). High-fat diets, a primary trigger of NAFLD, impair intestinal barrier integrity and promote endotoxin translocation. This activates inflammatory pathways in hepatocytes and promotes the secretion of inflammatory cytokines, thereby accelerating NAFLD progression and formation of an inflammatory microenvironment, while exacerbating hepatic lipid accumulation. (Wang Y. et al., 2023). As a novel class of “prebiotics,” LBPs improve the liver microenvironment of NAFLD by regulating the gut microbiota composition. When normal mice were given LBPs by gavage for 4 weeks, it significantly reduced TC, TG, and LDL in the serum and liver parenchyma of mice without affecting body weight. It also decreased interleukin-6 and increased interleukin-10, suggesting protective effects against fatty liver and systemic inflammation (Liang et al., 2023). In NAFLD models, LBPs further reduced serum TC, TG, LDL, AST, ALT, and alkaline phosphatase, while decreasing the levels of tumor necrosis factor alpha and interleukin-6, thereby improving hepatic inflammation (Li et al., 2022). By contrast, LBPs repolarize M2-type to M1-type macrophages, improve the immunosuppressive tumor microenvironment, and inhibit breast cancer metastasis (Liu et al., 2024). Therefore, LBPs may theoretically inhibit liver metastasis in NAFLD-related colorectal cancer, which is confirmed by our *in vivo* data.

Disruption of gut microbiota homeostasis and impairment of intestinal barrier function are key pathogenic drivers of NAFLD and major risk factors for CRLM(Qu et al., 2023; Wang R. et al., 2023; Shen et al., 2024). LBPs regulate gut microbiota by increasing microbial diversity, enhancing the abundance of beneficial genera—particularly *Bacteroides* and *Lactobacillus*—and promoting the production of SCFAs, including acetate, propionate, and butyrate (Liang et al., 2023). SCFAs inhibit colorectal cancer progression and improve liver lipid metabolism and represent the main metabolites generated during LBPs fermentation by intestinal microorganisms (Rekha et al., 2024). Using pseudo-germ-free models and fecal microbiota transplantation, our study confirmed that gut microbiota is the critical mediator of inhibitory effects of LBPs on NAFLD-related CRLM. Microbiota analysis revealed that LBPs intervention significantly increased diversity and richness in NAFLD mice. At the phylum level, *Bacteroidetes* abundance significantly increased following LBPs intervention. *Bacteroides* species efficiently metabolize plant polysaccharides, supplying nutrients and vitamins to both the host and microbial community while inhibiting cancer progression (Qu et al., 2025). At the genus level, LBPs increased *Cryptobacteroides, Evtepia,* and *Bacteroides H*. Notably, *Cryptobacteroides* abundance was negatively correlated with TC, TG, LDL, AST, and ALT levels and positively correlated with HDL levels. *Cryptobacteroides* is enriched in the gut microbiota of populations in Africa with agricultural or semi-agricultural and high-fiber diets, and the same trend has been observed in the population of the Malaysian archipelago (Jamaluddin et al., 2025; Maghini et al., 2025). The genus *Cryptobacteroides* has a higher density of carbohydrate-active enzymes genes, with enrichment in SCFAs biosynthetic pathways (Xu et al., 2024). *Polyporus umbellatus* polysaccharides, another plant polysaccharide, significantly increased the abundance of *Cryptobacteroides* in the gut microbiota of mice. These findings suggest that *Cryptobacteroides* may be a key taxon in LBPs catabolism and SCFAs production, consistent with our observation that LBPs significantly increased colonic butyrate levels.

Our findings further demonstrated that serum acylcarnitine levels were significantly elevated in NAFLD mice. Acylcarnitines are intermediates of mitochondrial fatty acid *β*-oxidation in hepatocytes, and impaired *β*-oxidation in NAFLD leads to their systemic accumulation. Elevated acylcarnitine levels are associated with increased risk of CRC (Downie et al., 2025), gastric cancer (Li et al., 2022), and lung cancers (Qi et al., 2021), and accumulation within gallbladder cancer cells promotes hepatic metastasis (Yang et al., 2024). During progression from NAFLD to hepatocellular carcinoma, long-chain acylcarnitines accumulate in the liver and activate the signal transducer and activator of transcription 3 signaling, facilitating tumorigenesis (Fujiwara et al., 2018). Moreover, exogenous acylcarnitines induce metastasis-associated phenotypes in breast cancer cells (Chen et al., 2025). These observations suggest that an acylcarnitine-rich hepatic microenvironment may promote CRLM in patients with NAFLD.LBPs increase colonic SCFAs production, enhance hepatic fatty acid *β*-oxidation, and ameliorate NAFLD (Li et al., 2022; Liang et al., 2023; Rekha et al., 2024). In the present study, LBPs treatment significantly elevated fecal butyrate levels while concomitantly reducing circulating acylcarnitine concentrations. Butyrate, a well-established histone deacetylase inhibitor, upregulates peroxisome proliferator-activated receptor alpha, thereby promoting hepatic fatty acid *β*-oxidation and alleviating NAFLD (Sun et al., 2018). Enhanced β-oxidation accelerates acylcarnitine metabolism in hepatocytes, alleviating lipid accumulation. Collectively, we propose that LBPs enhance hepatic fatty acid *β*-oxidation via the gut microbiota–butyrate axis, thereby improving impaired lipid metabolism in NAFLD and reducing acylcarnitine lipid accumulation in the body.

Fibroblast growth factor 21 *(FGF21)* is a metabolic regulator that promotes fatty acid oxidation (Trusz, 2024). Paradoxically, elevated serum FGF21 levels are associated with poor prognosis in colorectal, thyroid, and breast cancers (Kang et al., 2019; Florea et al., 2021; Ranuncolo et al., 2025). In breast cancer, the overexpression of *FGF21* in tumor tissues correlates with adverse clinical outcomes, and under NAFLD conditions, upregulated FGF21 acts as a mediator that promotes breast tumorigenesis (Sui et al., 2024). FGF21 is also markedly overexpressed in lung cancer, where it enhances cellular proliferation and migration via the *SIRT1/PI3K/AKT* signaling pathway (Yu et al., 2021). Excessive free fatty acids accumulated in the livers of NAFLD (Geng et al., 2021; Li et al., 2020)) can be taken up by colorectal cancer cells, thereby activating the Adenosine 5′-monophosphate-activated protein kinase (AMPK) signaling pathway, promoting mitochondrial fatty acid *β*-oxidation, and enhancing tumour stemness (Wen et al., 2017). AMPK activation induces upregulation of FGF21 expression (Grabacka et al., 2016). Mechanistically, the lipid-rich microenvironment within NAFLD-affected liver tissue induces elevated *FGF21* transcription levels in colorectal cancer cells, thereby promoting fatty acid β-oxidation and facilitating liver metastasis of colorectal cancer.Our findings indicate that prophylactic LBPs administration activates the gut microbiota-butyrate axis, thereby enhancing fatty acid *β*-oxidation in hepatocytes and consequently reducing the accumulation of free fatty acids within the liver. This metabolic remodeling diminishes the lipid-rich microenvironment that otherwise promotes *FGF21* upregulation in metastatic CRC cells, ultimately suppressing liver metastasis.

This study provides the first evidence that LBPs exert a gut microbiota–dependent preventive effect on NAFLD-related CRLM, addressing a critical gap in current knowledge. Nevertheless, several limitations should be acknowledged. First, additional *in vivo* functional studies are required to confirm whether butyrate is the principal effector metabolite mediating the anti-metastatic effects of LBPs. Second, the hypothesis that fatty acids promote the metastasis of colorectal cancer cells through the *FGF21-PI3K-AKT* signaling axis lacks direct experimental evidence and needs to be further clarified through subsequent functional rescue or pathway blocking experiments.It should also be noted that this represents a single-dose exploratory investigation. However, based on an extensive literature search, it was found that administering 200 mg/kg to mice improved the progression of NAFLD while demonstrating safety.

## 5 Conclusion

In conclusion, this study demonstrates that LBPs significantly prevent CRLM in a NAFLD-associated mouse model. The protective effects are mediated through the regulation of the gut microbiota and associated metabolic pathways. These findings indicate the potential of LBPs as preventive medications against CRLM through microbiota-targeted interventions.

## Data Availability

The data presented in the study are deposited in the NCBI BioProject repository, accession numbers PRJNA1357105 and PRJNA1354979.

## Glossary

CRLM: Colorectal Liver Metastasis
NAFLD: Non-alcoholic Fatty Liver Disease
LBPs: Lycium barbarum Polysaccharide
FMT: Fecal Microbiota Transplantation
CRC: Colorectal Cancer
16S rRNA: 16S Ribosomal Ribonucleic Acid
RNA-seq: RNA Sequencing
IHC: Immunohistochemistry
PCR: Polymerase Chain Reaction
UPLC: Ultra-High Performance Liquid Chromatography
SDS-PAGE: Sodium Dodecyl Sulfate–Polyacrylamide Gel Electrophoresis
BCA: Bicinchoninic Acid
ANOVA: Analysis of Variance
OTU: Operational Taxonomic Unit
PCoA: Principal Coordinates Analysis
LDA: Linear Discriminant Analysis
GO: Gene Ontology
KEGG: Kyoto Encyclopedia of Genes and Genomes
GSEA: Gene Set Enrichment Analysis
SCFAs: Short-Chain Fatty Acids
FGF21: Fibroblast Growth Factor 21
PI3K: Phosphoinositide 3-Kinase
AKT: Protein Kinase B
EMT: Epithelial-Mesenchymal Transition
AMPK: Adenosine 5‘-monophosphate-activated protein kinase
TC: Total Cholesterol
TG: Triglyceride
LDL: Low-Density Lipoprotein
HDL: High-Density Lipoprotein
AST: Aspartate Aminotransferase
ALT: Alanine Aminotransferase
FBS: Fetal Bovine Serum
H&E: Hematoxylin-Eosin
HPLC: High-performance liquid chromatography
AMPK: Adenosine 5’-monophosphate-activated protein kinase.
