# Supplementary material for: Lycium barbarum polysaccharides as prebiotics prevent colorectal cancer liver metastasis in non-alcoholic fatty liver disease by modulating gut microbiota–FGF21-PI3K-AKT axis
Source: Front Pharmacol. 2026 Mar 13;17:1735434. doi: 10.3389/fphar.2026.1735434 (PMC13021886; doi:10.3389/fphar.2026.1735434)
Supplement: Supplementary file 1 [file DataSheet1.pdf]

Supplementary Material

Supplementary Figures

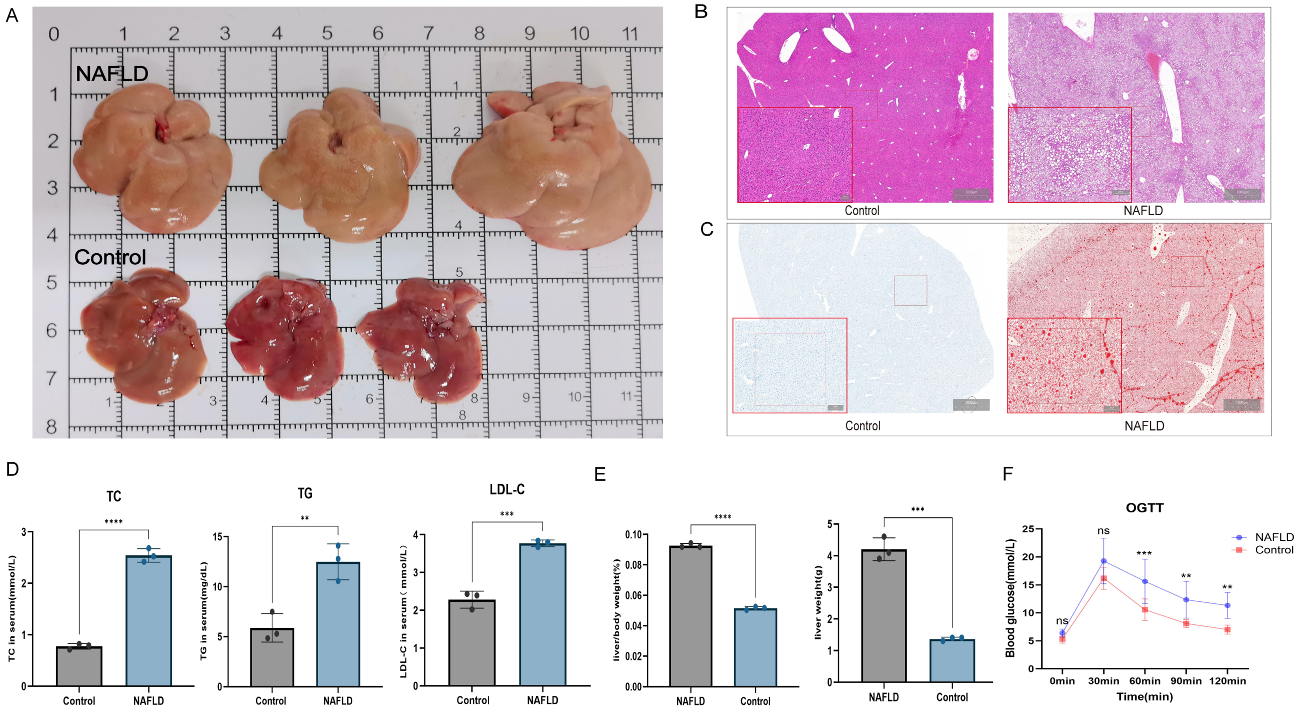

Supplementary fig：Validation of the NAFLD mouse model（**A**）Liver parenchymal images of the Control group and the NAFLD group（**B**）H&E staining of two groups of liver tissues（**C**）Oil Red O staining of two groups of liver tissues（**D**）Biochemical analysis was conducted to determine the levels of total cholesterol (TC), triglycerides (TG), and low-density lipoprotein cholesterol (LDL-C) in mouse serum（**E**）Comparison of liver weights and liver-to-body weight ratios between the two groups（**F**）Blood glucose levels of two groups of mice at different time points. $^{*}p < 0.05$, $^{**}p < 0.01$, $^{***}p < 0.001$, $^{****}p < 0.0001$.
